# Molecular Epidemiology and Genetic Variation of Pathogenic *Vibrio parahaemolyticus* in Peru

**DOI:** 10.1371/journal.pntd.0002210

**Published:** 2013-05-16

**Authors:** Ronnie G. Gavilan, Maria L. Zamudio, Jaime Martinez-Urtaza

**Affiliations:** 1 Instituto de Acuicultura, Universidad de Santiago de Compostela, Campus Universitario Sur, Santiago de Compostela, Spain; 2 Instituto Nacional de Salud, Jesus Maria, Lima, Peru; Universidad Peruana Cayetano Heredia, Peru

## Abstract

*Vibrio parahaemolyticus* is a foodborne pathogen that has become a public health concern at the global scale. The epidemiological significance of *V. parahaemolyticus* infections in Latin America received little attention until the winter of 1997 when cases related to the pandemic clone were detected in the region, changing the epidemic dynamics of this pathogen in Peru. With the aim to assess the impact of the arrival of the pandemic clone on local populations of pathogenic *V. parahaemolyticus* in Peru, we investigated the population genetics and genomic variation in a complete collection of non-pandemic strains recovered from clinical sources in Peru during the pre- and post-emergence periods of the pandemic clone. A total of 56 clinical strains isolated in Peru during the period 1994 to 2007, 13 strains from Chile and 20 strains from Asia were characterized by Multilocus Sequence Typing (MLST) and checked for the presence of Variable Genomic Regions (VGRs). The emergence of O3:K6 cases in Peru implied a drastic disruption of the seasonal dynamics of infections and a shift in the serotype dominance of pathogenic *V. parahaemolyticus*. After the arrival of the pandemic clone, a great diversity of serovars not previously reported was detected in the country, which supports the introduction of additional populations cohabitating with the pandemic group. Moreover, the presence of genomic regions characteristic of the pandemic clone in other non-pandemic strains may represent early evidence of genetic transfer from the introduced population to the local communities. Finally, the results of this study stress the importance of population admixture, horizontal genetic transfer and homologous recombination as major events shaping the structure and diversity of pathogenic *V. parahaemolyticus*.

## Author Summary

Infections caused by *Vibrio parahaemolyticus* have increased significantly over the last two decades, with cases now regularly reported globally. The emergence of cholera at global scale has brought the attention toward other *Vibrio* diseases in developing countries. This was the situation in Peru, where the investigation of *V. cholerae* in hospitals and regional public health laboratories after the dramatic emergence of cholera epidemic in 1991 enabled the identification of other pathogenic *Vibrio* throughout the whole country. The submission of all these bacteria to the Instituto Nacional de Salud (INS, Lima, Peru) for characterization generated an extraordinary repository of records and isolates which have been decisive for sizing the impact of *V. parahaemolyticus* infections on the population. The present study addresses, for first time, the impact of the arrival of a non-endemic population of *V. parahaemolyticus* on the genetic structure and virulence attributes of local populations. The detection of the pandemic clone of *V. parahaemolyticus* to Peru in 1997 changed not only the epidemic dynamics of this pathogen, but also the population structure and genetic variation of native populations through population admixture, horizontal genetic transfer and homologous recombination between native and introduced populations of pathogenic *V. parahaemolyticus*.

## Introduction

*Vibrio parahaemolyticus* is a Gram-negative halophilic bacterium that naturally inhabits marine and estuarine environments throughout the world. While many strains of *V. parahaemolyticus* are strictly environmental, some groups are pathogenic and may cause gastroenteritis in human [1]. *V. parahaemolyticus* is the leading human pathogen of bacterial food-borne diseases associated with the consumption of raw or undercooked seafood [2].

Recently, *V. parahaemolyticus* has gained notoriety due to global dissemination of infections [3]. The rise of infections was initially linked to the emergence of gastroenteritis throughout Asia associated with a single clone of the O3:K6 serovar in 1996 [4]. The subsequent detection of this clone causing infections in Peru [5], [6], and Chile [7] in 1997 indicated the pandemic expansion of O3:K6 clone infections. Afterwards, gastroenteritis cases associated with the O3:K6 clone were reported in many other countries and different areas of the world, such as the United States, Russia, Mozambique, Mexico, and Spain [3]. In addition to rapid dissemination of the pandemic clone, a parallel rise of human cases associated with other genetic groups has been reported in recent years. Specific genetic groups have been described as being associated with infections in particular geographic regions of the world. Consequently, the presence of local clones and serovars prevail among clinical cases, such as O4:K12 in the Pacific coast area of the United States [8], O4:K8 in the Peruvian coast [5], O4:K13 in Africa [9], and O4:K11 in the northeast of Spain [10].

The majority of clinical cases of *V. parahaemolyticus* have been associated with strains bearing the thermostable direct hemolysin (tdh) and/or TDH-related hemolysin (trh). Therefore, the presence of one or both hemolysins has been considered to be a conventional marker of *V. parahaemolyticus* virulence [11], [12]. However, it has been reported recently that not only the presence of hemolysins but also other virulence factors such as the type III secretion system (T3SS) have been involved in the cytotoxicity and enterotoxicity of *V. parahaemolyticus* [13], [14]. Whole genome sequencing of the clinical *V. parahaemolyticus* strain RIMD2210633 revealed the presence of two sets of genes encoding two different T3SSs, named T3SS1 and T3SS2, distributed in each chromosome [15]. A functional analysis of these two T3SSs revealed that T3SS1 is involved in cytotoxic activity, while T3SS2 has been related to enterotoxicity [13]. Consequently, the presence of T3SS2 has been consistently associated with pathogenicity of *V. parahaemolyticus* in humans. The T3SS2 found in the second chromosome of *V. parahaemolyticus* (T3SS2α) is part of a large pathogenicity island (PAI) of approximately 80 kb that also includes the *tdh*-genes. In the *trh*-positive strain TH3996, a novel PAI that is inserted in a distinctive pathogenic island containing a homologous T3SS2 (T3SS2β) has recently been described [16].

Comparative genome analysis of *V. parahaemolyticus* predicted that RIMD2210633 pathogenesis is associated with the presence of eight pathogenicity islands (VpaI) [17]. However, to date, VpaI that include T3SS2 have only been functionally characterized in infection models [13], [16]. Genomic analyses also revealed that different types of pathogenicity islands and mobile elements are the major structural differences between *trh*-positive and *tdh*-positive strains, including the pandemic clone [18].

*Vibrio parahaemolyticus* has not been routinely investigated in Peru and infections caused by this organism are rarely reported to the surveillance system. The mandatory investigation of *V. cholerae* in clinical laboratories after the dramatic emergence of cholera epidemic in 1991 in Peru contributed to the identification of other pathogenic *Vibrio* species. *Vibrio* strains isolated from hospitals and regional public health laboratories throughout the country were shipped to the Instituto Nacional de Salud (INS, Lima, Peru) for final identification and characterization. This extraordinary repository has been decisive for identification and evaluation of the impact of *V. parahaemolyticus* infections on the population, especially in remote regions and small villages along the Peruvian coast where seafood consumption constitutes the nutritional base of population.

The arrival of the pandemic clone to Peru in 1997 resulted in a major shift in the epidemic dynamics of *V. parahaemolyticus* in the region, replacing the seasonal and local self-limited infections attributed to native genetic groups by the generalization of infections exclusively caused by pandemic strains distributed across the country [5], [6]. Due to the environmental nature of *V. parahaemolyticus*, this overturn in population dominance and the subsequent population admixture may be expected to lead to an unpredictable impact on populations of pathogenic *V. parahaemolyticus* in Peru. To test this hypothesis, we investigated the population genetics, genomic variation and pathogenic islands distribution in a complete collection of non-pandemic strains recovered from clinical sources in Peru over the pre- and post-emergence of the pandemic clone. Pandemic and non-pandemic clinical strains representative of the entire period of study were subjected to serotyping and genotyping analysis by Multilocus Sequence Typing (MLST) and Variable Genomic Region (VGRs) analysis.

## Materials and Methods

### Epidemiological Data and Bacterial Strains

Peruvian *V. parahaemolyticus* strains were recovered from the collection of the National Reference Laboratory for Enteropathogens at the Instituto Nacional de Salud (INS) in Peru. This collection comprised 46 strains from the previously studied period of 1994 to 2005 [6] and 10 additional strains from the period of 2006 to 2007. To extend the comparison, a Chilean group, comprised of 13 strains from Antofagasta and Puerto Montt [19], [7], and 20 Asiatic strains, corresponding to 18 from Japan, one from Bangladesh and one from Thailand [20], [21], were included in the analysis (Table 1). The reference strain to *V. alginolyticus* ATCC17749 was also added to the panel of strains.

**Table 1:** Characteristics of *V. parahaemolyticus* strains included in this study.

Country	Strain	Isolation Year	Serovar	tdh	trh	GS-PCR	Source	ST	Reference
Peru	324-95, 326-95, 267-95	1995	O4:K8	+	−	−	Stool	88	[5]
	288-95	1995	O5:KUT	+	−	−	Stool	89	[5]
	090-96, 212-96	1996	O4:K8	+	−	−	Stool	**265**	[5]
	763-97, 906-97	1997	O3:K6	+	−	+	Stool	3	[5]
	3435-98, 784-98	1998	O3:K6	+	−	+	Stool	3	[5]
	275-99	1999	O3:K58	+	−	+	Stool	3	[5]
	276-99, 278-99, 279-99, 698-99	1999	O3:K6	+	−	+	Stool	3	[5]
	357-99	1999	OUT:KUT	−	−	−	Stool	19	[5]
	776-00	2000	O6:KUT	+	−	−	Stool	93	[5]
	330-00, 405-00, 429-00, 430-00, 461-00, 462-00, 511-00, 512-00	2000	O3:K6	+	−	+	Stool	3	[5]
	463-01	2001	O1:K33	−	−	−	Stool	94	[5]
	056-01, 498-01, 565-01, 568-01	2001	O3:K6	+	−	+	Stool	3	[5]
	vp196-02	2002	O4:K8	+	−	−	Env.	**265**	[5]
	004-02, 020-02, 169-02, 240-02, 551-02, 552-02, 553-02	2002	O3:K6	+	−	+	Stool	3	[5]
	038-03, 039-03, 131-03, 302-03,	2003	O3:K6	+	−	+	Stool	3	[5]
	205-05	2005	O1:KUT	+	−	+	Stool	3	[5]
	155-05, 156-05	2005	O1:KUT	−	+	−	Stool	65	[5]
	691-05	2005	O4:K8	+	−	−	Stool	**265**	[5]
	232-06	2006	O4:K8	+	−	−	Stool	**265**	This study
	301-07, 371-07,	2007	O1:KUT	+	−	+	Stool	3	This study
	304-07	2007	O3:K30	+	−	+	Stool	3	This study
	369-07, 438-07	2007	O3:KUT	+	−	+	Stool	3	This study
	437-07	2007	O3:KUT	+	+	−	Stool	64	This study
	1257-07, 1262-07, 245-07	2007	O4:K8	+	−	−	Stool	**265**	This study
Chile	ATC210, ATC220	1998	O3:K6	+	−	+	Stool	3	[21]
	PMC48.4	2004	O3:K6	+	−	+	Stool	3	[21]
	PMC39.5, PMC42.5	2005	O3:K6	+	−	+	Stool	3	[21]
	PMC29.7, PMC41.7, PMC55.7, PMC73.7	2007	O3:K6	+	−	+	Stool	3	[19]
	PMC53.7	2007	O3:K59	−	−	−	Stool	28	[19]
	PMC38.7	2007	O10:K20	+	−	−	Stool	63	[19]
	PMC60.7	2007	O1:KUT	+	+	−	Stool	64	[19]
	PMC75.7	2007	O1:KUT	−	+	−	Stool	65	[19]
Japan	ATCC17802	1951	O1:K1	−	+	−	Stool	1	[21]
	RIMD2210633	1996	O3:K6	+	−	+	Stool	3	[15]
	AQ3857	1983	O1:K1	+	+	−	Stool	83	[20]
	AQ3860	1983	O6:K46	+	+	−	Stool	83	[20]
	AQ4405	1989	O3:K72	+	+	−	Stool	83	[20]
	AQ4433	1989	O1:KUT	+	+	−	Stool	83	[20]
	AQ4704	1992	O1:KUT	+	+	−	Stool	83	[20]
	AQ4969	1994	O1:KUT	+	+	−	Stool	83	[20]
	AQ4966	1994	O1:KUT	+	+	−	Stool	83	[20]
	AQ4781	1992	O1:KUT	+	+	−	Stool	84	[20]
	AQ4815	1993	O1:K59	+	+	−	Stool	84	[20]
	AT4	1987	O4:K37	−	+	−	Env.	85	[20]
	AQ4889	1993	O4:K12	+	+	−	Stool	86	[20]
	AQ3810	1983	O3:K6	+	−	−	Stool	87	[20]
	AQ3855	1983	O6:K18	+	+	−	Stool	90	[20]
	AQ4901	1993	O3:K6	−	+	−	Stool	91	[20]
	KX-V132	1995	O3:KUT	+	+	−	Stool	92	[20]
	AQ4037	1985	O3:K6	−	+	−	Stool	96	[20]
Thailand	275	1990	O1:K1	+	+	−	Stool	82	[20]
Bangladesh	U5474	1980	O3:K6	+	−	−	Stool	14	[21]

### Detection of Genetic Markers and Serotyping

DNA extraction of *V. parahaemolyticus* strains was performed using an overnight culture in trypticase soy broth at 37°C using the Chelex-100 method [22]. Strains were confirmed by the presence of the *V. parahaemolyticus* species-specific genes *toxR* as described previously [23]. Additionally, the presence of the genes *tdh* and *trh* was determined according to procedures described by Tada et al. [24]. Group-specific PCR for the detection of the *toxRS* sequence of strains belonging to the pandemic clone of *V. parahaemolyticus* was performed as previously described [6], [25]. Serotyping Lipopolysaccharide (O) and capsular (K) serotypes were determined by a commercially available antisera scheme (Denka Seiken Corp., Tokyo, Japan).

### Genetic Relatedness and Population Structure

MLST analysis was performed as previously described [21], based on internal fragments of seven housekeeping genes: *recA*, *gyrB*, *dnaE*, *dtdS*, *pntA*, *pyrC*, and *tnaA*. Sequences of both strands were determined by custom sequencing (Macrogen Inc., Seoul, South Korea). All chromatograms were assembled, manually edited and trimmed in Bionumerics 5.1 (Applied-Maths, Kortrijk, Belgium). Allele numbers were assigned to each strain by comparing the nucleotide sequence at each locus to all known corresponding alleles available at the *V. parahaemolyticus* MLST Database (http://pubmlst.org/vparahaemolyticus/). Novel sequence variants and sequence types (ST) were deposited in this database, and ST assignment was performed using the MLST website tools (http://pubmlst.org). Nucleotide sequences of MLST locus corresponding to STs generated from the study were also deposited in GenBank under accession numbers KC542949–KC543109.

Population genetic relationships among *V. parahaemolyticus* strains included in this study were performed based on the MLST allelic profiles by a minimum spanning tree analysis implemented in BioNumerics 5.1 software (Applied-Maths, Sint Maartens-Latem, Belgium). Strains were grouped according to priority rules adopted from the BURST algorithm [26], with the highest priority given to profiles with the largest numbers of single locus variants or double-locus variants in the case of a match. Clonal complexes were defined as groups with a maximum neighbor distance of one change and a minimum size of two strains.

### Characterization of *V. parahaemolyticus* Variable Genomic Regions

Variable genomic regions (VGRs) were identified by comparative genome analysis based on a complete genome sequence (RIMD2210633) and two draft genomes of *V. parahaemolyticus* (AQ4037 and AQ3810) retrieved from the NCBI database (http://www.ncbi.nlm.nih.gov/). Genomic sequence data were analyzed by MUMmer 3.0 [27] to identify non-redundant genomic sequences. The identified sequences were listed as VGRs and used in further analyses. To determine the presence and distribution of VGRs among the strains included in this study, we carried out 31 different PCR assays using specific primers targeting the 23 regions identified by comparative analysis (Table 2). The PCR assays were performed in a 50-µl reaction volume containing 10 ng of genomic DNA, 1 U of Taq DNA polymerase (Roche, Mannheim, Germany), 0.2 µM of each primer (Sigma-Aldrich, Sigma-Aldrich, St. Louis, MO), and 200 µM of each deoxynucleoside triphosphate (Roche, Mannheim, Germany). PCR cycling conditions consisted of an initial denaturation step at 94°C for 3 min followed by 30 cycles of denaturation at 94°C for 50 s, 50 s at the annealing temperature, which was variable for each region (see Table 2), and extension step at 72°C for 1 min. A final extension step consisted of 10 min at 72°C.

**Table 2:** Primers used and *V. parahaemolyticus* genomic regions investigated.

ID	Chromose	Genome reference	Gene target	Primer Sequence (5′ - 3′)	Product size (bp)	Annealing T° (Extension time)	Associated genetic region
HGT1	chr1	RIMD 2210633	VP0390	TTTTCATCCATATCCATCGTCC	579	62 (1 min)	(VPaI-1)
			VP0392	GCTTCCTCTTACCCTTTTCA			
HGT1A			VP0389	AAGCGGAGTCGTGTTTTTGTGT	1723	64 (2 min)	
			VP0392	TCCTGCCTTCGCGCTATCTTTG			
HGT2	chr1	RIMD 2210633	VP1072	AAAGAGTTGACCGCTGTAAGG	1677	62 (1.3 min)	(VPaI-3)
			VP1073	CGCTGTAACGTCATTGGTCTA			
HGT3	chr1	RIMD 2210633	VP1394	ACGGCACCATCAGATTCATACCA	1116	62 (1.3 min)	T6SS
			VP1396	ATAGCACCGCCCAATAGACCTGT			
HGT3A			VP1408	GAGATAACCGCACAACCAAG	1065	62 (1.3 min)	
			VP1409	CTGATCAAAAAACCGAGGCT			
HGT4	chr1	RIMD 2210633	VP1561	GAGCGCTTCAAAGTGGGGATTAC	1142	62 (1.3 min)	Phage f237
			VP1563	CTGCCATTGGATTGATAGAAACC			
HGT5	chr1	RIMD 2210633	VP1796	TGGGCTGAAAACAATAACCTCT	1026	62 (1.3 min)	Super Integron
			VP1798	TGCTGCAGCCAAACTAATACC			
HGT5A			VP1845	TCGATCATAGGCCGCATAACAG	1293	62 (1.3 min)	
			VP1848	TTCGCATCCAACTTCATCAACC			
HGT6	chr1	RIMD 2210633	VP1886	TGGGCTACGTCTTTGCTACTGA	1577	62 (1 min)	cold-shock proteins,
			VP1888	ATACCATGATGCCAAAGAGACG			Ribonuclease R
HGT6A			VP1889	TGGTGGTGAAGACTTGTTTG	594	62 (1 min)	
			VP1890	TCTTTCTCTGGCTCGTCATT			
HGT7	chr1	RIMD 2210633	VP2139	GAAGCGCGTTGGCAGGAATAC	1066	64 (1.3 min)	
			VP2142	TGGAAGGGGGACGGATACGAT			(VPaI-4)
HGT8	chr1	RIMD 2210633	VP2903	AAAGAAAGGTGGGAGGGTGAAG	753	62 (1 min)	(VPaI-5)
			VP2905	TTTGACAAGGAGAACGGAGGTG			
HGT9	chr2	RIMD 2210633	VPA0434	ATCTCAAACCCCCGCTCAAAAG	770	62 (1 min)	putative site-specific
			VPA0435	TAAAAGACACCGCCTACGACTGC			recombinase
HGT10	chr2	RIMD 2210633	VPA1261	GTTTTGCCACAGCCATTATTTC	963	62 (1 min)	(VPaI-6)
			VPA1262	TTCCCTTTCGATCTCCAACACC			
HGT12	chr1	RIMD 2210633	VP0639	GGGCATGTTAAAGCGTTAGT	948	62 (1 min)	(VPaI-2)
			VP0641	TAGGTAAGTGTATCGAGCGT			
HGT13	chr1	RIMD 2210633	VP0657	ACAGCGGCACAGAATATTAC	792	62 (1 min)	type IV pilin
			VP0658	GTTGGCTCGTTTAATTTGGT			
HGT14	chr1	RIMD 2210633	VP1365	AGGTTGTGAAAGGGGATTAG	1010	62 (1.3 min)	hypothetical proteins
			VP137	GACAACAAACAACGAGACCA			
HGT15	chr1	RIMD 2210633	VP2693	AACAATACGACGATCAGGAC	856	62 (1 min)	MSHA pilin
			VP2695	TACACCACCGACAGGAATCA			
HGT16	chr2	RIMD 2210633	VPA0891	AGCCAGAATACCCAAAGGGA	1065	64 (1.3 min)	Phage related genes
			VPA0893	GCCACCAAACGAAAACACA			
HGT16A			VPA0904	GGAAACGGTGGCTTTTTAATG	1051	62 (1.3 min)	
			VPA0908	GAGCTTTGAGGGAGTGAAAT			
HGT17	chr2	RIMD 2210633	VPA0953	CTCTACTAATTCAACGACCACT	766	62 (1 min)	biofilm-associated surface
			VPA0954	CATATCTTTGCGGGTCATCT			proteins
HGT18	chr2	RIMD 2210633	VPA1564	TAACCAAAAGAGCGCAAAAG	859	62 (1.3 min)	hypothetical protein
			VPA1565	AACATCAAAACCAACACCAG			
HGT19	chr2	RIMD 2210633	VPA1702	TCAAAGTGGTCGCAGCATAA	1026	62 (1.3 min)	hypothetical protein
			VPA1704	AAGACCAATGGCGAAGAAA			
HGT20	chr1	AQ3810	A79_3286	GGAAGCGAACAACATGAGAT	922	62 (1.3 min)	Type I restriction system
			A79_3288	GAATGAAGAAATAGGTGAGGG			
HGT20A			A79_3276	CCACTTCTGCACATCTTCAA	627	62 (1 min)	
			A79_3277	GCATTTTTCTCTCGCCCATT			
HGT21	chr1	AQ3810	A79_2542	CACCCTAAGCATATCAAACA	701	62 (1 min)	Integron cassette
			A79_2543	GACAGCAGAAGAAATATCCA			
HGT22	chr1	AQ3810	A79_5185	ATAGTCAGGTTTTCATCGGG	941	62 (1 min)	hypothetical protein
			A79_5186	TTAAGGAAGAGGGGAAAGCA			
T3SS2α	chr2	RIMD 2210633	VPA1338	CGTCGTGGCGTGATCTCTACTT	1083	64 (1 min)	T3SS2α (VPaI-7)
			VPA1339	ATTGCAGCAGCCATTACGAACA			
			VPA1355	TTTAACCCCAACGATTCAGTGC	709	62 (1 min)	
			VPA1356	CATGCGTAGTCAAGCCCGTTAT			
T3SS2β	chr2	AQ4037,	vscN	TTGGGGTGATTTCGACTTTT	767	60 (1 min)	T3SS2β
		TH3996	vscC	GTGGCGTTTATGATCTTGTT			
			vopD2	GACGCCAATGAAATACCAAA	1068	60 (1 min)	
			vopB	CAGAAGAAGGCAGAACAAAA			

### Evolutionary Analysis

Phylogenetic relationships were inferred using ClonalFrame v1.1 software [28]. MLST loci sequences of unique STs were input into ClonalFrame using the default options. Two independent ClonalFrame runs were performed consisting of 500,000 iterations. The first 100,000 iterations in each run were discarded, and the phylogeny and additional model parameters were sampled every 100 generations in the last 400,000 iterations. The phylograms sampled from the two different runs were concatenated and summarized in a 50% majority rule consensus tree constructed by ClonalFrame GUI [28]. The convergence of the Markov Chain Monte Carlo (MCMC) in both runs was proven based on the Gelman-Rubin test as implemented in ClonalFrame [29].

To visualize potential associations between the phylogeny of housekeeping genes and the distribution of VGRs, we performed a transversal clustering analysis. For this purpose, the concatenated sequences of 67 strains were grouped by the mean of their phylogenetic relationship using ClonalFrame (50% majority consensus), while the VGR data were grouped using a UPGMA algorithm mean of their value (0 = absent and 1 = present), resulting in a data matrix ordered according to both phylogenetic relationship and VGR clustering.

## Results

### Epidemiology Trends and Laboratory Investigation of *V. parahaemolyticus*

**Figure 1:**
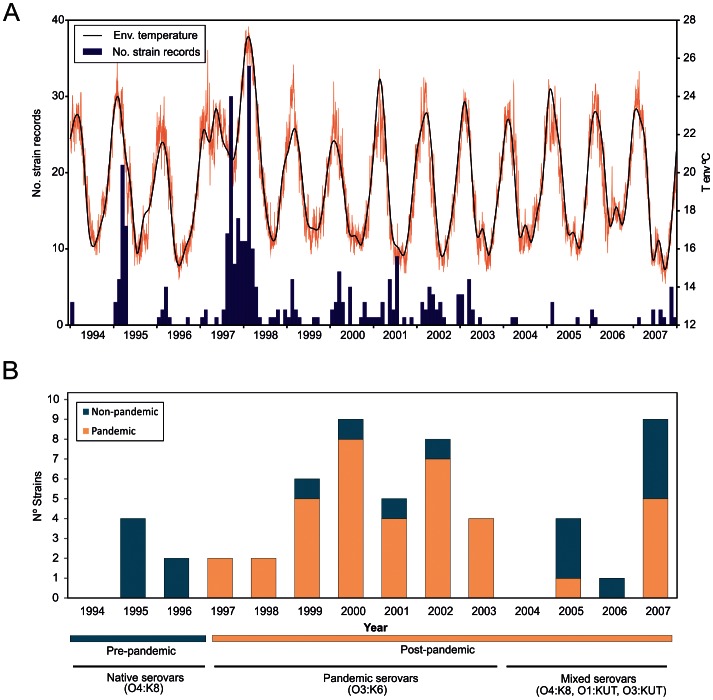
Distribution of *V.* parahaemolyticus over the study period. (A) Number of *V. parahaemolyticus* strains received at the National Institute of Health in Peru between 1994 and 2007 and environmental temperature over the same period. (B) Rate of pandemic versus non-pandemic strains over the study period; information on the dominant serotypes of each period is provided below the X-axis.

A total of 324 *V. parahaemolyticus* strain records were retrieved from the INS database from 1994 to 2007. These strains were obtained from gastroenteritis cases that occurred in different regions of Peru and were subsequently submitted to the INS in Lima for identification and storage. The overall distribution of *V. parahaemolyticus* cases over the 14 years of study (Figure 1) showed a characteristic seasonal pattern with annual peaks of incidence concurring with the warmest months. This epidemic pattern was uniquely disturbed in the course of the austral winter of 1997 when *V. parahaemolyticus* cases dramatically increased coincidently with an anomalous rise of temperature. The highest incidence of cases was observed from July 1997 to May 1998 with two peaks during September 1997 and February 1998. The epidemic dynamics were restored from 1999 onwards.

From the 324 cases recorded over the period 1994–2007, only 56 *V. parahaemolyticus* strains could be recovered from the INS collection. Distinct serovar dominances were detected over different periods of the study. From 1994 to 1996, infections were associated with serovars O4:K8 and O5:KUT. This serovar dominance abruptly changed during the winter of 1997, with the emergence of infections caused by strains belonging to the pandemic clone. Finally, an undefined pattern of serovar dominance was detected after the emergence of the pandemic clone in 1997–98. In this post-pandemic period, pandemic strains were identified together with O4:K8 strains as well as multiple serovars not previously detected (O1:K33, O1:KUT, O3:K30, O3:K58, O3:KUT, O5:KUT, O6:KUT and OUT:KUT). Pandemic strains represented the largest group of strains (n = 38, 67.9%) detected from 1997 onwards, and O3:K6 was the most frequent serovar among the pandemic strains (n = 31, 55.4%), although serovars O3:K58 and O3:KUT were also identified in the pandemic group. Serovar O4:K8 was the second most frequent group of strains over the whole period (n = 11, 19.6%), whereas the remaining serovars (n = 14) represented 25% of the total strains.

### Genetic Structure of *V. parahaemolyticus*

**Figure 2:**
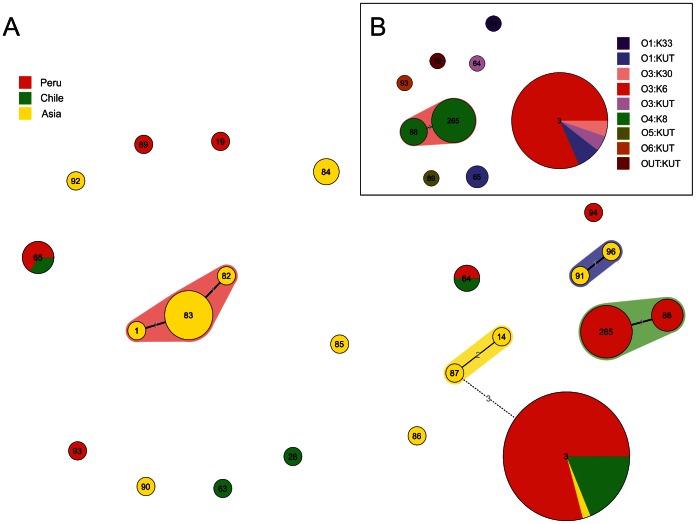
Minimum spanning tree (MST) of *V.* parahaemolyticus. A) MST of 89 MLST allelic profiles of the whole dataset included in this study. The colors are based on the origin of isolation. B) MST of the Peruvian subset that includes 56 strains. The colors are based on the serotyping group. Each circle represents an MLST genotype, and its size is proportional to the number of strains. The numbers along the branch links of MLST genotypes denote the amount of allelic differences.

The population genetic structure of *V. parahaemolyticus* strains representative of Peru, Chile, and Asian countries was analyzed by MLST (Fig. 2). MLST profiles of *V. parahaemolyticus* (n = 89) were categorized into 23 sequence types (STs). The recA gene of strains included within the ST265 (n = 8, O4:K8 serotype) showed an unexpected large PCR product of approximately 1500 bp (773 bp in the original) with a nucleotide identity diverging 18–19% from the characteristic sequence of *V. parahaemolyticus*.

The Peruvian group was split into 9 different STs (Fig. 2B). Minimum spanning tree analysis identified a single clonal complex consisting of ST88 (n = 3) and ST265 (n = 8) both of which included O4:K8 strains differing in a single locus. All of the strains belonging to the pandemic complex were grouped in ST3 (n = 24), whereas the remaining strains were included in 6 unrelated STs. Minimum spanning tree of the 23 STs resulting from the MLST analysis of the 89 strains showed a similar topography and group discrimination. Peruvian, Chilean and Asian pandemic strains shared the sequence of the 7 loci and were assigned to a single ST (ST3). Strains from Peru and Chile shared two additional STs: ST65 composed by O1:KUT strains and ST64 including strains belonging to O3:KUT and O1:KUT serovars. Three other clonal complexes (CC) were identified among trh+ Asian strains: one CC included STs 1, 83 and 82; a second group consisted of ST96 and ST91; and a third CC was shared by STs 87 and 14. The remaining strains were clustered in different and unrelated STs with differences in more than 5 loci.

### Evolutionary History

**Figure 3:**
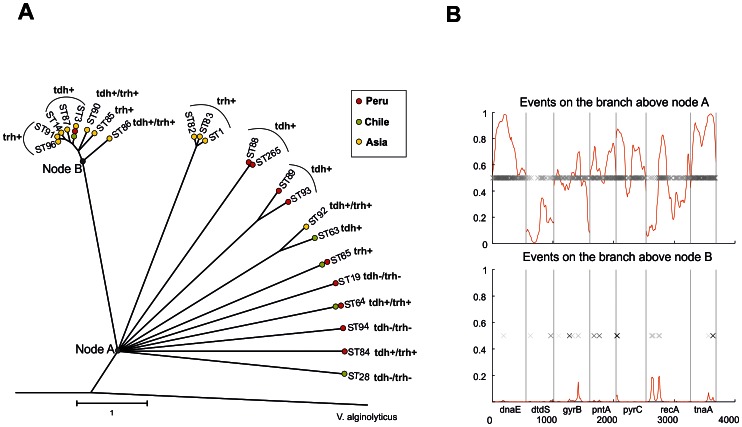
Phylogenetic tree and recombination events. A). The phylogenetic relationship of 22 *V. parahaemolyticus* STs was generated using ClonalFrame by 50% majority-rule consensus visualized using MEGA4. *V. alginolyticus* was used as an outgroup. Nodes A and B correspond to the recombination events presented in Figure 4B. Red, green and yellow circles denote the place of isolation from Peru, Chile and Asia, respectively. Coalescent units are indicated by the scale bar. B) ClonalFrame representation of the recombination event probability occurring in MLST concatenated is represented on the *x*-axis with the red line indicating the probability at each locus for an import event on a scale from 0 to 1 (*y*-axis). Each inferred substitution in the graph is represented by a cross, the intensity of which indicates the posterior probability for that substitution.

The nucleotide sequences of 23 unique STs identified among the *V. parahaemolyticus*, as well as homologous sequences of *V. alginolyticus* (outgroup), were concatenated, and their phylogenetic relationships were inferred by ClonalFrame. The clonal genealogy inferred from the data revealed a star-like topology of *V. parahaemolyticus* delineated into two evident lineages, with the rest of the STs remaining unresolved (Figure 3). The pandemic lineage consisted of all of the pandemic strains from Asia, Chile and Peru (ST3), as well as other STs that included Asian pre-pandemic strains of diverse serotypes and the three variants of hemolysin-related genotypes. A second lineage consisted of trh+ strains of STs 1, 82 and 83, all of them from Asia.

The genealogy showed the influence of recombination on the genetic diversification of *V. parahaemolyticus* with a central node (node A) from which all of the branches diverged. This specific topology indicated an unresolved phylogeny due to the non-identification of a common ancestor. ST88 and ST265, comprising all the O4:K8 strains were grouped in a single lineage. ST89 and ST93, which included Peruvian strains, also shared a common linage, as well as ST92 and ST63, sequence types composed of Asian and Chilean strains, respectively. For each branch of the reconstructed genealogy, ClonalFrame identified fragments that were likely imported. The influence of mutation and recombination events in the generation of polymorphisms was further investigated in the two cluster nodes (A and B) indicated in Figure 3A. Node A showed a high probability of importing events consistent with recombination substitutions, while divergence in node B, which included the Pandemic lineage, most likely originated as a result of point mutations (Fig. 3B). ClonalFrame analysis shows that the relative impact of recombination versus point mutation expressed as a ratio (r/m) was approximately 1.45, and that the relative frequency of recombination in comparison to point mutation (ρ/θ) was approximately 0.20.

### VGR Distribution

**Figure 4:**
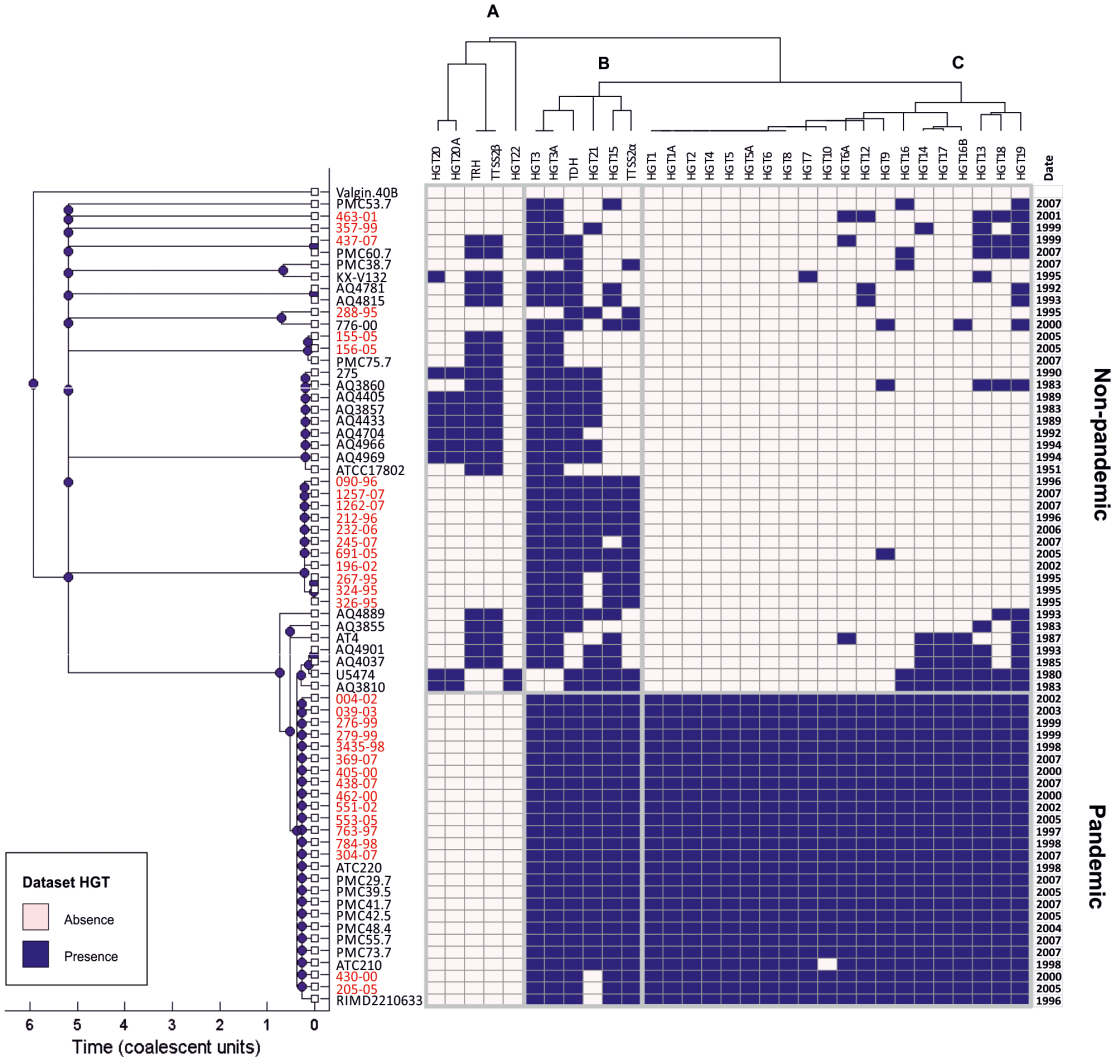
Transversal clustering of phylogenetic relationships and genomic variation. Clustering was built using a ClonalFrame tree of MLST allele sequences of 67 *V. parahaemolyticus* strains, while the VGR data were grouped using a UPGMA algorithm, resulting in a data matrix ordered according to both phylogenetic relationship and the prevalence of VGRs. Solid boxes and open boxes in the appropriate column indicate VGR presence or absence, respectively. Peruvian subset names are shown in red.

To extend our analysis of the phylogenetic relationships and to understand the impact of genomic variation in the evolutionary history of *V. parahaemolyticus*, the distribution of VGRs was assessed by PCR assays in the 67 strains. A transversal clustering analysis was performed to visualize the evolutionary significance of the presence of genomic regions within the strain collection (Fig. 4).

ClonalFrame majority-rule consensus tree building with the whole set of strains revealed the same group structure as resolved by the clonal genealogy. However, analysis of VGR data showed a specific clustering of VGRs with three different groups (A, B, and C) (Fig. 4). Cluster A grouped five different genomic regions almost exclusive for trh+ strains with the exception of two tdh+ strains (U5474 and AQ3810). The presence of TTSS2β was only detected in strains bearing the *trh* gene. This cluster also included regions HGT20 and HGT22 encoding for the Type I restriction system, which was only identified in *trh*+ Asian strains. Cluster B included 6 genomic regions; two of them (HGT3 and HGT3A) correspond to ORFs coding the type 6 secretion system (T6SS1), which were present in all of the clinical strains with the exception of 4 *tdh*+/*trh*− non-pandemic strains isolated in Peru, Chile, and Asia. T3SS2α was singularly detected in those strains with genotype *tdh*+/*trh*− but was not present in those strains positive for the *tdh* and *trh* gene. Cluster C consisted of the majority of VGRs (20 genomic regions), which were uniformly distributed among almost all the pandemic strains. Some of these regions were partially present in all Asian strains phylogenetically related to pandemic strains, while those were less frequent in the remaining non-pandemic strains and distributed with an undefined pattern. Non-pandemic Peruvian strains showed a shift in the distribution of the VGRs included within this cluster from the pre-pandemic period to 1999 and afterwards. Genomic regions such as HGT6A (ribonuclease R), HGT12 (VPaI2), HGT9 (site-specific recombinase), HGT14 (hypothetical protein), HGT16B (phage genes), HGT13 (type IV pilin), HGT18 (hypothetical protein) and HGT19 (hypothetical protein), which are characteristic of pandemic strains, were exclusively identified in those non-pandemic strains from Peru isolated after the arrival of the pandemic clone in 1997. Finally, none of the VGRs investigated were found in *V. alginolyticus* ATCC17749.

## Discussion

*Vibrio parahaemolyticus* represents an intriguing food-borne pathogen and poses a significant threat to public health in Peru. However, despite this concern for public health, the knowledge of the molecular epidemiology and genetic structure of this pathogen remains incomplete.

Epidemiology of *V. parahaemolyticus* in Peru have been historically associated with sporadic outbreaks linked with seafood consumption. Since 1983, clinical strains were dominated by the presence of local serovars such as O4:K8 that was isolated from both clinical and environmental samples [30]. These infections were characteristically related to auto-limiting outbreaks detected along the coastal regions over summer months. This epidemic pattern shifted in 1997 with the unexpected arrival of pandemic *V. parahaemolyticus* to Peru [5]. The presence of the O4:K8 serovar in pre- and post-pandemic periods allowed for the identification of this group as the dominant population among clinical strains in Peru and the cause of recurrent outbreaks during the warmest months [31], [5]. The totality of strains recovered from infections in 1997 and 1998 belonged exclusively to the pandemic clone. After the period of the infections associated with the pandemic clone in 1997–98, the dominance of this clone in clinical infections began to decline and a mix of different serovars began to emerge. This specific epidemiological trend of arrival and rapid decline of pandemic *V. parahaemolyticus* infection in Peru clearly contrasts with the infection dynamics found in Chile, the neighboring country where infections associated with the pandemic clone started first in 1997 and subsequently in 2004, and where the pandemic clone has dominated the clinical isolations since the second epidemic radiation in 2004 [32]. The arrival of the pandemic clone to Peru provided a unique opportunity for testing the potential impact of an introduced genetic group on the structure and genetic variability of local pathogenic populations. One particular feature of the epidemiology of *V. parahaemolyticus* in Peru after the appearance of the pandemic clone was the sudden apparition of diverse serovars not detected previously. Serovars O1:K33, O1:KUT, O3:K30, O3:K58, O3:KUT, O5:KUT, O6:KUT and OUT:KUT had not been isolated prior to 1998. Similar results were found in a previous retrospective study carried out in Peru covering the 1993–2002 period [6], which reported the presence of serovars O3:K68, O3:K58, OUT:K6, O6:K18, O11:KUT, O11:K15 and OUT:KUT after 1998. The flourishing of serovars and genotypes in Peru may be related to the particular vehicle for the introduction and propagation of pandemic strains. The epidemic dissemination of this pandemic clone along the coast of Peru corresponded with the expansion and dynamics of the poleward propagation and the receding of tropical waters linked to the 1997 El Niño event [5], [33].

Another important aspect to be considered to understand the possible genetic impact of the pandemic clone on Peruvian local populations is the comparative analysis of phylogeny inferred from MLST sequences and the distribution of variable regions in the panel of strains. The pattern of VGR distribution forming cluster C showed a conserved presence for all regions among strains belonging to the pandemic clone, which provides an additional evidence for the highly clonal nature of this phylotype [34], [18]. On the contrary, a sparse presence of VGRs in cluster C was observed, showing an undefined pattern among non-pandemic strains. A detailed analysis of variations and phylotypes showed that VGRs in cluster C are only present in strains isolated after the arrival of the pandemic clone in 1997. This specific pattern of distribution and the connection between these strains and the arrival of the pandemic clone may suggest a common origin for all of these groups. However, the presence of a region coding hypothetical genes (VPA0434 and VPA0435) in one O4:K8 strain also raises the possibility of a local horizontal transfer from pandemic strains to local *Vibrio* communities in Peru.

Horizontal gene transfer appears to be the major force shaping both the genomic variation and virulence of *V. parahaemolyticus*, as evidenced in previous studies linking the acquisition of pathogenicity islands with the emergence of the pandemic clone [17], [18]. The results of transversal clustering analysis revealed that the presence of T3SS, T6SS and mannose-sensitive hemagglutinin (MSHA) pilus were clustered together in most clinical isolates showing a clear association with the pathogenicity of these strains, suggesting that these regions are conserved in pathogenic strains and could be a good marker of pathogenicity.

The evolutionary history of pathogenic lineages of *V. parahaemolyticus* has been analyzed previously by different approaches [21], [17], [35]. However, the previous analysis of population structure was not sufficiently integrated within the epidemic dynamics prevailing in a specific region so that there could be an adequate evaluation of the shift in genotype and population dominance over different periods. ClonalFrame genealogy inferred a star-shaped tree with long terminal branches showing a primary diversification affecting all of the STs, likely as a result of recombination events [36]. A defined lineage grouped all of the pandemic strains as well as most of the Asian genotypes showing a hierarchical pattern recently evolved from a common ancestor, likely due to the course of successive point mutations. The overall results evidenced the influence of recombination events in the diversification of most pathogenic *V. parahaemolyticus* genotypes.

Homologous recombination in housekeeping genes has been found naturally in *Vibrio*, and it is an important driver of diversification in this genus [37]. ClonalFrame analyses of the whole concatenated dataset showed rates of recombination of 1.45 and 0.20 for r/m and ρ/θ, respectively. These data suggest an intermediate rate of recombination among the strains characterized [38]. This estimate of recombination frequency suggests that recombination is relatively rare compared to other species, such as *Streptococcus uberis* (ρ/θ, 9.0) [39] and *Clostridium perfringens* (ρ/θ, 3.2) [40], but it is approximately similar to that observed for other groups, such as lineage I of *Listeria monocytogenes* (ρ/θ, 0.13) [41], This particular feature may be related to the exclusive use of pathogenic strains in the study, which are characterized by a clonal diversification [38].

To conclude, the results of this study describe the epidemiological impact caused by the introduction of the pandemic clone in Peru on the epidemiology and structure of the local population of *V. parahaemolyticus*. The presence of genomic regions characteristic of the pandemic clone in other non-pandemic strains provides early evidence of genetic transfer from the introduced population to the local communities. Additionally, the genetic relationships based on MLST and VGR analyses support the epidemiological connection between pandemic and non-pandemic strains isolated in both Peru and Chile. The phylogenetic and genomic analysis performed allowed us to determine the recent origin of the pandemic clone lineage, probably caused by successive acquisition of genomic regions, as well as the influence of recombination events in the diversification of non-pandemic pathogenic *V. parahaemolyticus*. Ultimately, these results provide a preliminary framework about evolutionary history of *V. parahaemolyticus*. Recent advances in high throughput sequencing are revolutionizing the field of population genetics of human pathogens. Application of fine-scale analysis based on whole genome sequences in future studies of pathogenic bacteria will contribute to improve our knowledge of the epidemic dynamics and routes of dispersion of *Vibrio* diseases [42], [43].
